# OsWOX13: a molecular bridge linking Ehd1 to florigen Hd3a in regulating rice heading date

**DOI:** 10.1093/plcell/koag270

**Published:** 2026-09-07

**Authors:** Hongwei Jing

**Affiliations:** Assistant Features Editor, The Plant Cell, American Society of Plant Biologists; Department of Horticultural Science, North Carolina State University, Raleigh, NC 27695, United States

In cereal crops, heading date marks the transition from vegetative to reproductive development and is a major determinant of grain yield and environmental adaptation. The heading date transition is predominantly controlled by photoperiod, with day length providing the key environmental signal that regulates the onset of heading (Zong et al. 2024). In rice (*Oryza sativa* L.), the floral integrators Heading date 3a (Hd3a) and RICE FLOWERING LOCUS T1 (RFT1) are central to the photoperiodic pathways regulating heading date. These proteins act as florigens, mobile signals produced in leaves and translocated to the shoot apical meristem triggering the floral transition. Upstream regulation of these florigens occurs via 2 distinct pathways: the *GIGANTEA-Heading date 1-Heading date 3a/RFT1* (*GI-Hd1-Hd3a/RFT1*) pathway, and the *EARLY HEADING DATE 1* (*Ehd1*)-*Hd3a/RFT1* pathway (Song et al. 2024). The *WUSCHEL Related Homeobox* gene *OsWOX13* has been reported to promote heading date, but the molecular and genetic mechanisms underlying its function remain largely unknown. Elucidating how OsWOX13 integrates upstream heading signals and regulates downstream florigen genes will provide important insights into the regulatory network controlling heading date in rice.

In a recent study, Ming Yu and colleagues (Yu et al. 2026) identified OsWOX13 as a key signal transducer that links the heading integrator *Ehd1* to the florigen gene *Hd3a*, thereby ensuring precise regulation of rice heading date (Fig. 1). To identify direct targets of Ehd1, the authors performed ChIP-Seq using *Ehd1-FLAG* overexpression plants and detected a prominent Ehd1-binding peak within the P2 region of the *OsWOX13* promoter. Additional in vivo and in vitro assays confirmed that Ehd1 directly binds the *OsWOX13* promoter and activates its expression. Genetic and mutant studies further revealed that *OsWOX13* acts downstream of *Ehd1* to regulate heading date. Subsequent promoter analysis showed that *Hd3a* is the only candidate target gene harboring the previously characterized OsWOX13-binding motif (CAATCAAT). Consistent with this regulatory relationship, *OsWOX13* and *Hd3a* exhibited overlapping spatiotemporal expression patterns and similar diurnal rhythms in leaves.

**Figure 1:**
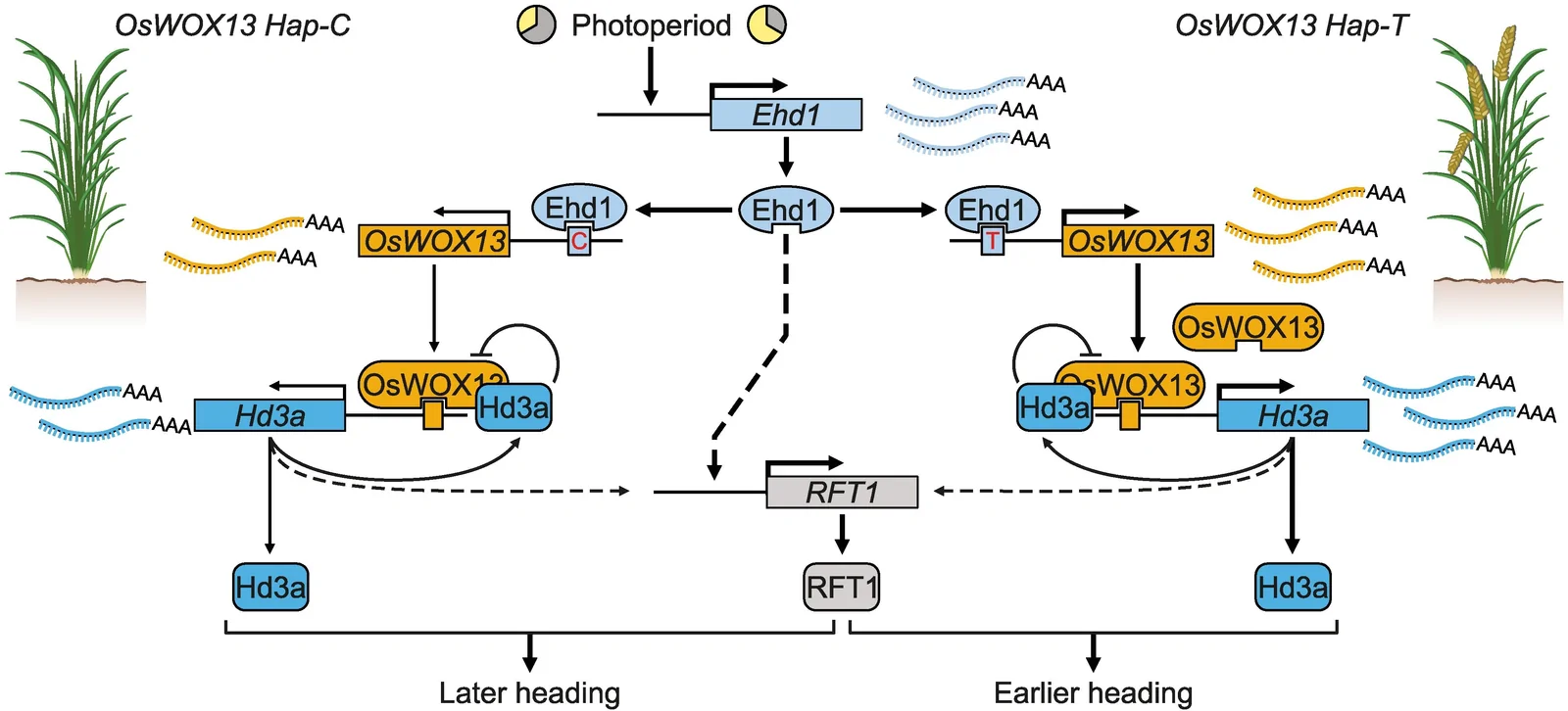
Proposed model of the *Ehd1-OsWOX13-Hd3a* regulatory pathway controlling heading date in rice. *EARLY HEADING DATE 1* (*Ehd1*) activates the expression of the *WUSCHEL Related Homeobox* transcription factor *OsWOX13*, with stronger transcriptional activation in the *Hap-T* haplotype than in *Hap-C*. OsWOX13 directly binds the *HEADING DATE 3a* (*Hd3a*) promoter to activate its transcription. The Hd3a protein, in turn, interacts with OsWOX13 to attenuate its transcriptional activation activity, thus fine-tuning *Hd3a* expression and indirectly regulates *RICE FLOWERING LOCUS T1* (*RFT1*). As a result, the higher expression of *OsWOX13* in the *Hap-T* haplotype leads to increased florigen production and promotes earlier heading in rice. Adapted from Yu et al. (2026), Fig. 8.

The authors next investigated whether natural variation in *OsWOX13* contributes to differences in rice heading date. They identified a single-nucleotide polymorphism (SNP) in the *OsWOX13* promoter that defines 2 major haplotypes, *Hap-C* (homozygous for cytosine) and *Hap-T* (homozygous for thymine), with *Hap-T* associated with earlier heading. Functional analyses showed that the *Hap-T* promoter exhibited stronger transcriptional activity and greater binding by Ehd1 than the *Hap-C* promoter, resulting in higher *OsWOX13* and *Hd3a* expression and earlier heading. Conversely, replacing the *Hap-T* allele with *Hap-C* in the Longjing 31 background reduced *OsWOX13* and *Hd3a* expression and delayed heading. Interestingly, *Hap-T* was highly enriched in cultivated *Japonica* rice and was preferentially found in high-latitude regions, whereas *Hap-C* predominated in *Indica* rice and lower-latitude regions. Thus, natural variation in the *OsWOX13* promoter tunes the *Ehd1-OsWOX13-Hd3a* pathway and may have contributed to the geographic adaptation of cultivated rice.

Overall, this study identifies OsWOX13 as a molecular link between the heading-day integrator Ehd1 and the florigen Hd3a that fine-tunes rice heading date (Fig. 1). Despite these advances, several important questions remain. The authors noted that the *oswox13 ehd1* double mutant exhibited a heading date phenotype similar to that of the *ehd1* single mutant, suggesting that *Ehd1* regulates heading date through additional downstream effectors independent of *OsWOX13*. Future studies should focus on these downstream targets to determine how Ehd1 integrates diverse environmental and endogenous cues to coordinately regulate the florigen genes *Hd3a* and *RFT1*. It will also be important to elucidate the molecular basis by which OsWOX13 directly activates *Hd3a* transcription through promoter binding, whereas the Hd3a protein interacts with OsWOX13 to attenuate its transcriptional activity. Addressing these questions will deepen our understanding of the regulatory network controlling heading day and may facilitate the development of rice varieties better adapted to diverse environments.

## Recent related articles in *The Plant Cell*

- Jin and colleagues (Jin et al. 2026) identified a rice early-heading mutant and cloned the causal gene, *EARLY HEADING AT LONG DAY 4* (*ELD4*). They showed that ELD4 encodes a zinc finger transcription factor that physically interacts with the pseudo-response regulator OsPRR95 to directly regulate *OsMADS51* expression, thereby controlling heading date.
- Wu and colleagues (Wu et al. 2025) discovered that blue light receptor *FLAVIN-BINDING KELCH REPEAT F-BOX 1a* (*ZmFKF1a*) interacts with *GIGANTEA 1* (*ZmGI1*), stabilizing it and promoting its nuclear localization via a blue light–dependent mechanism to accelerate shoot apex development and flowering in maize.
- Yuan and colleagues (Yuan et al. 2025) identified a regulatory module in which the ubiquitin ligase SEVEN IN ABSENTIA 3 (SINA3) and the WUSCHEL HOMEOBOX 14 (WOX) transcription factor act to coordinately regulate multiple aspects of tomato (*Solanum lycopersicum*) development, including fruit growth, seed set, and fruit maturation.
- Zhang and colleagues (Zhang et al. 2023) identified *Early heading date 5* (*Ehd5*), which encodes a WD40 domain–containing protein that exhibits circadian rhythmic expression and functions as a positive regulator of flowering, highlighting an important role for the circadian clock in controlling heading date in rice.

## Data Availability

No new data were generated or analysed in support of this research.
